# Supplementary figures and images for: The complete mitogenome of Lysmata vittata (Crustacea: Decapoda: Hippolytidae) with implication of phylogenomics and population genetics
Source: PLoS One. 2021 Nov 4;16(11):e0255547. doi: 10.1371/journal.pone.0255547 (PMC8568142; doi:10.1371/journal.pone.0255547)

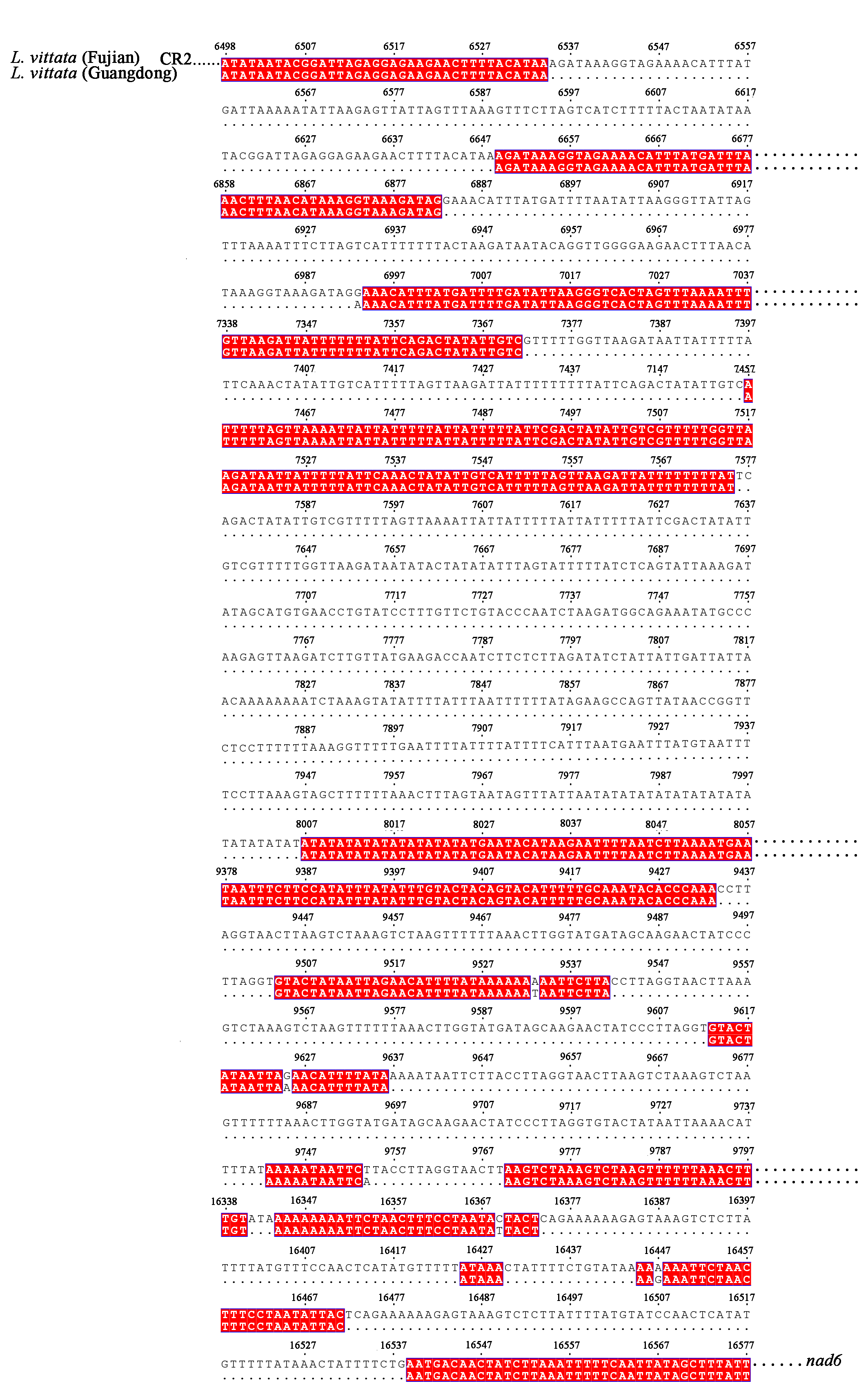

Supplement: S1 Fig — (TIF) [file pone.0255547.s001.tif]

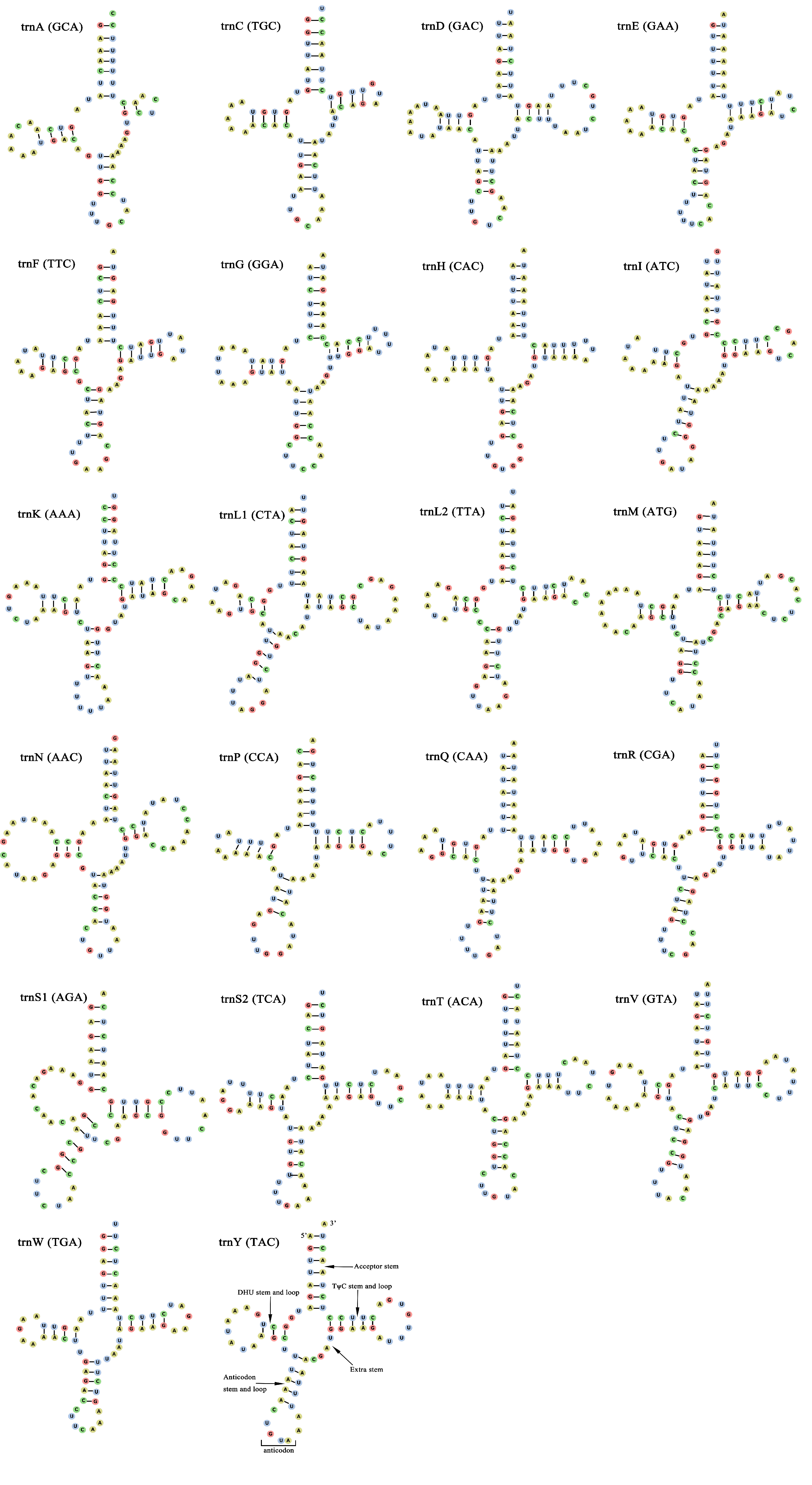

Supplement: S2 Fig — (TIF) [file pone.0255547.s002.tif]
