# Supplementary material for: The complete mitogenome of Lysmata vittata (Crustacea: Decapoda: Hippolytidae) with implication of phylogenomics and population genetics
Source: PLoS One. 2021 Nov 4;16(11):e0255547. doi: 10.1371/journal.pone.0255547 (PMC8568142; doi:10.1371/journal.pone.0255547)
Supplement: S1 Table — (DOC) [file pone.0255547.s003.doc]

**Supplementary Table 1: List of species used to construct the phylogenetic tree.**

| **Suborder/Superfamily** | **Family** | **Species** | **GenBank No.** |
| --- | --- | --- | --- |
| Caridea | Hippolytidae | *Lysmata vittata* (Fujian) | This study |
|  |  | *Lysmata vittata* (Guangdong) | MW285083.1 |
|  |  | *Lysmata boggessi* | MZ144584.1 |
|  |  | *Lysmata debelius* | MW691200.1 |
|  |  | *Lysmata amboinensis* | NC_050676.1 |
|  |  | *Exhippolysmata ensirostris* | MK681888.1 |
|  |  | *Thor amboinensis* | NC_051930.1 |
|  |  | *Saron marmoratus* | NC_050677.1 |
|  |  | *Lebbeus groenlandicus* | NC_045223.1 |
|  | Alpheidae | *Palaemon sinensis* | NC_045090.1 |
|  |  | *Palaemon serenus* | NC_027601.1 |
|  |  | *Palaemon capensis* | NC_039373.1 |
|  |  | *Macrobrachium rosenbergii* | NC_006880.1 |
|  |  | *Macrobrachium bullatum* | NC_027602.1 |
|  |  | *Macrobrachium lanchesteri* | NC_012217.1 |
|  | Alpheidae | *Alpheus inopinatus* | NC_041151.1 |
|  |  | *Alpheus japonicus* | NC_038116.1 |
|  |  | Alpheus hoplocheles | NC_038068.1 |
|  |  | *Alpheus distinguendus* | NC_014883.1 |
| Anomura | Paguridae | *Pagurus longicarpus* | NC_003058.1 |
| Brachyura | Portunidae | *Charybdis feriata* | NC_024632.1 |
|  |  | *Charybdis bimaculata* | NC_037695.1 |
|  |  | *Charybdis japonica* | NC_013246.1 |
|  |  | *Charybdis natator* | NC_036132.1 |
|  |  | *Scylla paramamosain* | MG197997.1 |
|  |  | *Scylla olivacea* | FJ827760.1 |
|  |  | *Scylla tranquebarica* | NC_012567.1 |
|  |  | *Scylla serrata* | NC_012565.1 |
|  |  | *Portunus trituberculatus* | NC_005037.1 |
|  |  | *Portunus pelagicus* | KT382858.1 |
|  |  | *Portunus gracilimanus* | NC_040124.1 |
|  |  | *Callinectes sapidus* | NC_006281.1 |
|  | Potamidae | *Geothelphusa dehaani* | NC_007379.1 |
|  | Varunidae | *Eriocheir japonica* | AY274302.1 |
|  |  | *Eriocheir sinensis* | NC_006992.1 |
|  |  | *Eriocheir hepuensis* | MK159104.1 |
|  | Eriphiidae | *Pseudocarcinus gigas* | NC_006891.1 |
| Penaeoidea | Penaeidae | *Penaeus monodon* | NC_002184.1 |
|  |  | *Fenneropenaeus chinensis* | NC_009679.1 |
|  |  | *Litopenaeus stylirostris* | NC_012060.1 |
|  |  | *Litopenaeus vannamei* | NC_009626.1 |
|  |  | *Farfantepenaeus californiensis* | NC_012738.1 |
| Sergestoidea | Sergestidae | *Acetes chinensis* | NC_017600.1 |
|  |  | *Sergia lucens* | NC_037043.1 |
| [Achelata](https://www.ncbi.nlm.nih.gov/Taxonomy/Browser/wwwtax.cgi?mode=Tree&id=6730&lvl=3&lin=f&keep=1&srchmode=1&unlock) | Palinuridae | *Panulirus stimpsoni* | NC_014339.1 |
|  |  | *Panulirus japonicus* | NC_004251.1 |
|  |  | *Sagmariasus verreauxi* | NC_022736.1 |
|  |  | *Panulirus versicolor* | NC_028627.1 |
|  |  | *Puerulus angulatus* | NC_041155.1 |
