# Supplementary material for: The complete mitogenome of Lysmata vittata (Crustacea: Decapoda: Hippolytidae) with implication of phylogenomics and population genetics
Source: PLoS One. 2021 Nov 4;16(11):e0255547. doi: 10.1371/journal.pone.0255547 (PMC8568142; doi:10.1371/journal.pone.0255547)
Supplement: S2 Table — (DOC) [file pone.0255547.s004.doc]

**Supplementary Table 2: Summary of *Lysmata vittata* mitogenome.**

| **Gene** | **Direction** | **Location** | **Size(bp)** | **Anticodon** | **Star**  **codon** | **Stop**  **codon** | **Intergenic**  **nucleotides** |
| --- | --- | --- | --- | --- | --- | --- | --- |
| CR1 | — | 1－650 | 650 | — | — | — | 0 |
| *trnI* | F | 651－717 | 67 | GAT | — | — | 92 |
| *trnQ* | R | 810－877 | 68 | TTG | — | — | 17 |
| *trnM* | F | 895－971 | 77 | CAT | — | — | 6 |
| *nad2* | F | 978－1982 | 1005 | — | ATG | TAA | 73 |
| *trnW* | F | 2056－2122 | 67 | TGA | — | — | 2 |
| *trnC* | R | 2125－2187 | 63 | GCA | — | — | 4 |
| *trnY* | R | 2192－2262 | 71 | CTA | — | — | 37 |
| *cox1* | F | 2300－3913 | 1614 | — | TTG | TAG | 0 |
| CR2 | — | 3914－7734 | 3821 | — | — | — | 0 |
| *trnL2* | F | 7735－7799 | 65 | TAA | — | — | 0 |
| CR3 | — | 7800－8687 | 888 | — | — | — | 0 |
| *cox2* | F | 8688－9380 | 693 | — | ATG | TAA | 483 |
| *trnK* | F | 9864－9934 | 71 | CTT | — | — | 3 |
| *trnD* | F | 9938－10011 | 74 | GTC | — | — | 0 |
| *atp8* | F | 10012－10176 | 165 | — | ATG | TAA | -7 |
| *atp6* | F | 10170－10844 | 675 | — | ATG | TAA | 41 |
| *cox3* | F | 10886－11641 | 756 | — | GTG | TAA | 18 |
| *trnG* | F | 11660－11728 | 69 | TCC | — | — | 0 |
| *nad3* | F | 11729－12082 | 354 | — | ATG | TAA | 39 |
| *trnR* | F | 12122－12190 | 69 | TCG | — | — | 13 |
| *trnA* | F | 12204－12263 | 60 | TGC | — | — | 149 |
| *trnN* | F | 12413－12489 | 77 | GTT | — | — | 81 |
| *trnS1* | F | 12571－12638 | 68 | TCT | — | — | 4 |
| *trnE* | F | 12643－12708 | 66 | TTC | — | — | -2 |
| *trnF* | R | 12707－12772 | 66 | GAA | — | — | 0 |
| *nad5* | R | 12773－14504 | 1732 | — | ATG | T | 0 |
| *trnH* | R | 14505－14570 | 66 | GTG | — | — | 0 |
| *nad4* | R | 14571－15906 | 1336 | — | ATG | T | -7 |
| *nad4L* | R | 15900－16145 | 246 | — | TTG | TAA | 53 |
| *trnT* | F | 16199－16266 | 68 | TGT | — | — | 2 |
| *trnP* | R | 16269－16335 | 67 | TGG | — | — | 209 |
| *nad6* | F | 16545－17048 | 504 | — | ATC | TAA | 0 |
| *cob* | F | 17049－18185 | 1137 | — | ATG | TAA | 155 |
| *trnS2* | F | 18341－18410 | 70 | TGA |  |  | 14 |
| *nad1* | R | 18425－19351 | 927 | — | ATT | TAA | 188 |
| *trnL1* | R | 19540－19610 | 71 | TAG | — | — | -23 |
| *rrnL* | R | 19588－21081 | 1494 | — | — | — | 4 |
| *trnV* | R | 21086－21157 | 72 | TAC |  | — | 23 |
| *rrnS* | R | 21181－22001 | 821 | — | — | — | — |
