# Supplementary material for: The complete mitogenome of Lysmata vittata (Crustacea: Decapoda: Hippolytidae) with implication of phylogenomics and population genetics
Source: PLoS One. 2021 Nov 4;16(11):e0255547. doi: 10.1371/journal.pone.0255547 (PMC8568142; doi:10.1371/journal.pone.0255547)
Supplement: Attachment — Submitted filename: Response to Reviewers-20210904.doc [file pone.0255547.s005.doc]

Reviewer #1: The manuscript presented the first record of mitochondrial genome of the shrimp Lysmata vittata (Crustacea: Decapoda: Hippolytidae) and used it together with mitogenomes of the close families from the GenBank for reconstruction of phylogenetic tree. The data is valuable but only one mitogenome sequence was included in this manuscript. Nowadays, sequencing and describing a mitogenome is routine work and I can not find the novelty of the manuscript. In the manuscript, authors only described the mitogenome sequence and did not compared its structure with close species. Also, authors stated that phylogeny of Hippolytidae is problematic but the study is unable to solve the existing problem. In addition, even though authors stated that “we are the first to publish the mitochondrial genome sequence of the Hippolytidae species in the GenBank database”, a number of mitogenome sequence of Hippolytidae and Lysmatidae are available in the GenBank, such as Lysmata boggessi (GenBank no. MZ144584), Lysmata debelius (GenBank no. MW691200), Saron marmoratus (GenBank no. MT795210) and Exhippolysmata ensirostris (GenBank no. MK681888). It is surprise that authors did not make comparison between L. vittata mitogenome with these species. Moreover, these species were not included in the phylogenetic tree reconstruction. Because of that, the finding and discussion of the manuscript are poor.

Response: Many thanks for your professional suggestions. According to your suggestion, we have added the structural comparison and phylogenetic analysis of *L. vittata* mitogenome and its relative species in this article. Please see lines 130-143, 214-225 and 240-257. Your suggestions are of great help to us in improving the quality of article.

Reviewer #2: This manuscript is written with minor grammatical errors. Please improve the manuscript again. The experimental design is properly set up and the problem statement is well defined. I would like to suggest the authors discuss and make a comparison with the available complete mitogenome of the same species, L. vittata from Guangdong province China. It seems that the size of the mitogenomes of both individuals is different even they were collected not far away from each other as Guangdong and Fujian are next to each other. The author should also include both individuals in the phylogenetic tree and the gene arrangement comparison. Apart from this, I also suggest the authors compare all the genes from the mitogenome of each individual and discuss. From this, authors can determine the individual variation of L. vittata from China. Please also improve the phylogenetic tree figures as the figures are quite blurry.

Response: Thanks for your suggestions. This manuscript has been revised by an English speaking person. In addition, we have compared and discussed *L. vittata* (Fujian) and *L. vittata* (Guangdong) mitogenome sequences and hope that it is now clearer. Please see lines 135-143. Apart from this, we have also improved the phylogenetic tree figures.

Reviewer #3: The manuscript is well designed and written, the results are sound and helpful for better understanding the mitogenome of Decapoda and for the phylogenetics. Here I have several concerns as below,

The authors should tell the readers why you carried out this study, is there any problem need to be solved in the Background section.
Response: Thank you for the suggestion. The purpose and meaning of this research have been added in the introduction section. Please see lines 32-40 and 56-65.

The authors analyzed the phylogenetic relationship under the order Decapoda, however, they introduce limited information in the Introduction section, I suggest the authors to add some information about crabs.
Response: We are grateful for the suggestion. The content of the introduction section has been revised. At present, there are relatively few studies on the complete mitogenomes of Hippolytidae species, so we have selected some species under the order Decapoda to participate in the phylogenetic analysis. The main purpose of our phylogenetic analysis is to understand the classification status of *L. vittata* in the Hippolytidae, and our analysis results also illustrate the monophyleticity of the genus *Lysmata* and controversy exists in the classification of some species of Hippolytidae. Therefore, there is no information about crabs in the introduction section, but we added crab information when we performed phylogenetic analysis.

At line 65, what do “fresh leaves” mean?
Response: We apologize for the writing error in the original manuscript. The "fresh leaves" has been revised in the text. Please see line 70.

I wonder for the phylogenetic analysis, did the authors used crabs? if not, they could not use Decapoda.

Response: Thank you for your precious advice. Those advice are all valuable and very helpful for revising and improving our paper. We have added crab data to the phylogenetic analysis content. Please see Supplementary Table 1.

We would like to thank the referee again for taking the time to review our manuscript.
