# Supplementary material for: The complete mitogenome of Lysmata vittata (Crustacea: Decapoda: Hippolytidae) with implication of phylogenomics and population genetics
Source: PLoS One. 2021 Nov 4;16(11):e0255547. doi: 10.1371/journal.pone.0255547 (PMC8568142; doi:10.1371/journal.pone.0255547)
Supplement: Attachment — Submitted filename: Response to Reviewers.doc [file pone.0255547.s006.doc]

Reviewer #1: Author’s efforts to improve the submitted manuscript following reviewer’s comments have been done. Therefore, the current version of manuscript is much better than previous one. However, similar to the first comment, the only addition of a single new mitogenome, which does not show special meaning of mtgenome structure, to a crab molecular phylogeny is still problem. In addition, one more crucial problem is that the authors should justify why authors construct the phylogenetic trees for the decapods and what phylogenetic problem authors want to solve in the article. If the authors would like to present a novel decapod tree more comprehensive families and taxonomic representatives should be included. If the authors want to know the phylogenetic position of the *Lysmata vittata*, the tree will be enough with related species and taxa of L. vittate. In spite of these comments the final decision from the editor charging the article will be respected.

Response: Many thanks for your professional suggestions. We found that the mitogenome structure of *L. vittata* is different from other species in the same genus. Part of the mitogenome of *L. vittata* has translocations. There are 3 CR regions, and the length of the genome sequence is about 7 kb longer than that of normal shrimp species. Therefore, this manuscript analyzes the special mitogenome structure of *L. vittata*. In addition, in the phylogenetic analysis, there are currently limited mitogenome data of Hippolytidae species (all available data was collected), we borrowed the mitogenome data of some species in the Decapoda to conduct phylogenomics and population genetics analysis. Although the dispute over the taxonomic status of Hippolytidae still cannot be resolved, we are making more efforts to resolve this dispute. The purpose of establishing phylogenetic tree is to determine the phylogenetic position of the *Lysmata vittata*. In order to make this manuscript more express the author's thoughts, we have revised the title of the article, called "The complete mitogenome of Lysmata vittata (Crustacea: Decapoda: Hippolytidae) with implication of phylogenomics and population genetics." Thanks again for your valuable comments on our manuscript.

Reviewer #3: The authors have revised the manuscript according my comments, so now I have no more comments. The authors have revised the manuscript according my comments, so now I have no more comments.

Response: Thank you for taking the time to review our manuscript.
